# Interleukin-19 in Breast Cancer

**DOI:** 10.1155/2013/294320

**Published:** 2013-04-22

**Authors:** Ying-Yin Chen, Chien-Feng Li, Ching-Hua Yeh, Ming-Shi Chang, Chung-Hsi Hsing

**Affiliations:** ^1^Department of Medical Research, Chi-Mei Medical Center, Tainan 710, Taiwan; ^2^Department of Anesthesiology, Chi-Mei Medical Center, Tainan 710, Taiwan; ^3^Department of Biotechnology, National Formosa University, Yunlin 632, Taiwan; ^4^Department of Pathology, Chi-Mei Medical Center, Tainan 710, Taiwan; ^5^Institute of Medical Science, College of Health Science, Chang Jung Christian University, Tainan 711, Taiwan; ^6^Institute of Biochemistry and Molecular Biology, Medical College, National Cheng Kung University, Tainan 701, Taiwan; ^7^Department of Anesthesiology, College of Medicine, Taipei Medical University, Taipei 110, Taiwan

## Abstract

Inflammatory cytokines within the tumor microenvironment are linked to progression in breast cancer. Interleukin- (IL-) 19, part of the IL-10 family, contributes to a range of diseases and disorders, such as asthma, endotoxic shock, uremia, psoriasis, and rheumatoid arthritis. IL-19 is expressed in several types of tumor cells, especially in squamous cell carcinoma of the skin, tongue, esophagus, and lung and invasive duct carcinoma of the breast. In breast cancer, IL-19 expression is correlated with increased mitotic figures, advanced tumor stage, higher metastasis, and poor survival. The mechanisms of IL-19 in breast cancer have recently been explored both *in vitro* and *in vivo*. IL-19 has an autocrine effect in breast cancer cells. It directly promotes proliferation and migration and indirectly provides a microenvironment for tumor progression, which suggests that IL-19 is a prognostic marker in breast cancer and that antagonizing IL-19 may have therapeutic potential.

## 1. Introduction

Breast cancer is one of the most common malignant tumors in women [1] and becoming the principal cause of cancer death. The etiology of breast cancer is multifactorial, and its clinical course and molecular and pathological features are highly diverse [2]. Estrogen receptor (ER), progesterone receptor (PR), and human epithelial growth factor receptor 2 (HER2) are recognized as common clinical tumor markers for tumor growth and progression and as indicators of determining appropriate therapy for patients with breast cancer [3]. A lack of these factors has consistently been associated with a poorer prognosis [4]. In addition to these three molecular markers, a growing body of evidence has associated inflammation with a poor prognosis and reduced survival in patients with breast cancer [5, 6]. Cytokines, one of the inflammatory mediators, are emerging as factors that may significantly affect the growth of tumors *in vivo* [7–10] and be important in breast carcinogenesis [7, 11–13]. The role of hematopoietic growth factors, interferons, lymphokines, chemokines, and cytokines in tumor microenvironments has been intensively studied [14–16], especially in breast cancer [7, 13, 17–19]. Cytokines have been implicated in the mechanism for tumor cell evasion of the immune surveillance system [20, 21].

Cytokines, which are low molecular weight pleiotropic glycoproteins, are secreted primarily by immune cells and affect many different adjacent target cells: they alter target-cell function and modulate cell death, growth, and differentiation at very low concentrations [2]. Cytokines produced by cancer tissue [11, 12] and the expression of interleukin (IL)-1*β*, IL-6, IL-8, IL-10, IL-12, and IL-19, monocyte-chemoattractant-protein (MCP-) 1, and macrophage-inflammatory-protein- (MIP-) 1*β* are upregulated in breast cancer [7, 13, 17, 19]. Cytokines are important intercellular mediators of angiogenesis and leukocyte infiltration in breast cancer, and they promote or inhibit the growth of breast cancer depending on their relative concentrations and the presence of other regulatory factors in the tumor microenvironment [2, 7]. Changes in the relative concentration of some cytokines, such as IL-1, IL-6, IL-11, and IL-19, and transforming-growth-factor- (TGF-) *β*, mediated both directly and indirectly by the tumor stimulate breast cancer proliferation and invasion [1, 7, 13]. Despite massive investment in biomarker discovery, only a few have been recognized as potential biomarkers and tested in clinical practice [7]. The need for robust and accurate diagnostic markers that predict responses to breast cancer therapy is urgent. We recently reported [13] that upregulated IL-19 in breast cancer promoted tumor progression and affected clinical outcome: increased IL-19 expression in invasive ductal carcinoma (IDC), human breast tissue was associated with tumor staging. Elevated IL-19 levels were also closely correlated with inflammatory cell components, which accounted for tumor progression and metastasis.

In this review, we focus on the crucial roles of IL-19 in breast carcinogenesis and highlight principal recent findings on the effects of IL-19 in clinical outcomes and mechanisms of tumorigenesis and metastasis. Additionally, the potential role of IL-19 for assessing tumor staging, tumor aggressiveness, and disease progression is discussed.

## 2. IL-19, a Recently Discovered IL-10 Family Cytokine, Modulates Inflammatory Diseases

IL-19 is found within the IL-10 family gene cluster: IL-10, -19, -20, -22, -24 (MDA-7), -26 (AK155), -28, and -29 [23–28]. An *α*-helical protein, IL-19 functions as a monomer and is structurally similar to IL-10, which contains a hydrophobic core held by two disulphide bonds formed by four specific positions of cysteine (Cys57–Cys109 and Cys58–Cys111) [29, 30]. Despite a sequence homology of up to 30% between 159 of their amino acids, the biological functions of IL-19 and IL-10 are distinct [27, 31–34]. Stimulating monocytes with granulocyte-macrophage colony-stimulating factor (GM-CSF), lipopolysaccharide (LPS), and other cytokines (IL-4, IL-6, IL-17, and TNF-*α*) induces them to express IL-19 [35, 36]. IL-19 spurs the production of IL-6 and TNF-*α* by monocytes [37] and the release of Th2 cytokines by T cells [38], signals through the IL-20R1/IL-20R2 heterodimeric receptor, which it shares with IL-20 and IL-24, but with a different binding affinity [39], and stimulates tyrosine phosphorylation and nuclear translocation of STAT3 [28, 40, 41]. IL-19 derived primarily from macrophages and, to a lesser extent, from B cells and nonimmune cells including epithelial cells, endothelial cells, skin keratinocytes, and fetal membranes [25, 42]. Many reports showed that IL-19 has proinflammatory effects [24, 27, 28, 37]. In contrast, IL-19 has an anti-inflammatory property identified in inflammatory bowel disease [43] and vascular inflammatory diseases [44]; this property directly induces hyporesponsiveness in CD4-positive (+) T cells and promotes their regulatory activity, which may contribute to immunosuppression in patients after cardiac surgery [45].

IL-19 contributes to a range of diseases and disorders, such as breast cancer [13], asthma [37], endotoxic shock [46], uremia [47], psoriasis [48], rheumatoid arthritis [49], and periodontal and vascular disease [24]. IL-19 expression in uremic patients is upregulated and correlated with Th2 immune responses that might be involved in cytokine dysregulation [47]. Induction of IL-19 occurs in severe sepsis [46] and postcardiopulmonary bypass patients with a parallel shift that corresponds to the changes in TNF-*α* and IL-6 [36]. Thus, the potentiation of IL-19 expression is recognized as important in the pathogenesis of systemic inflammatory diseases. In mice with endotoxic shock, IL-19 mRNA levels are elevated in brain, heart, lung, liver, and kidney tissues [46]. Upregulated IL-19 in sepsis induces lung and liver tissue injury by inducing apoptosis and the production of IL-6, IL-8, TNF-*α*, and reactive oxygen species (ROS) [46]. However, the effects of IL-19 on inflammatory bowel disease are therapeutic and significantly reduced innate-mediated colonic inflammation in murine model [43]. Its anti-inflammatory activity is also found in vascular inflammatory diseases: IL-19 attenuates the response to injury of vascular smooth muscle cells (VSMCs) by diminishing human antigen R-mediated proliferative and inflammatory mRNA transcripts [44] and promoting heme oxygenase-1-mediated ROS reduction [50]. Thus, IL-19 is a pleiotropic cytokine that can suppress or stimulate immune regulation and disease [24].

## 3. Cytokine IL-19 Is a Prognostic Marker in Breast Cancer

### 3.1. IL-19 Is Involved in Tumor Biology

IL-19 expression in tumor cells has been investigated using tissue microarray technology and an immunohistochemical survey with an anti-IL-19 monoclonal antibody in 15 neoplastic tissue types (Table 1) [22]. IL-19 protein was positively stained in several types of tumor cells, especially in squamous cell carcinoma (SCC) of the skin, tongue, esophagus, and lung and invasive duct carcinoma (IDC) of the breast [22]. SCC of the oral cavity also expressed IL-19 mRNA and its receptors (IL-20R1/R2). In addition, IL-19 specifically activated intracellular signals and induced proliferation in two cell lines (OEC-M1 and OC3) derived from SCC of the oral cavity. This study provides important references for further investigation of the biological functions and clinical implications of IL-19 in humans.

### 3.2. IL-19 Expression Is Correlated with Advanced Tumor Stage, Higher Metastasis, and Poor Survival

**Figure 1:**
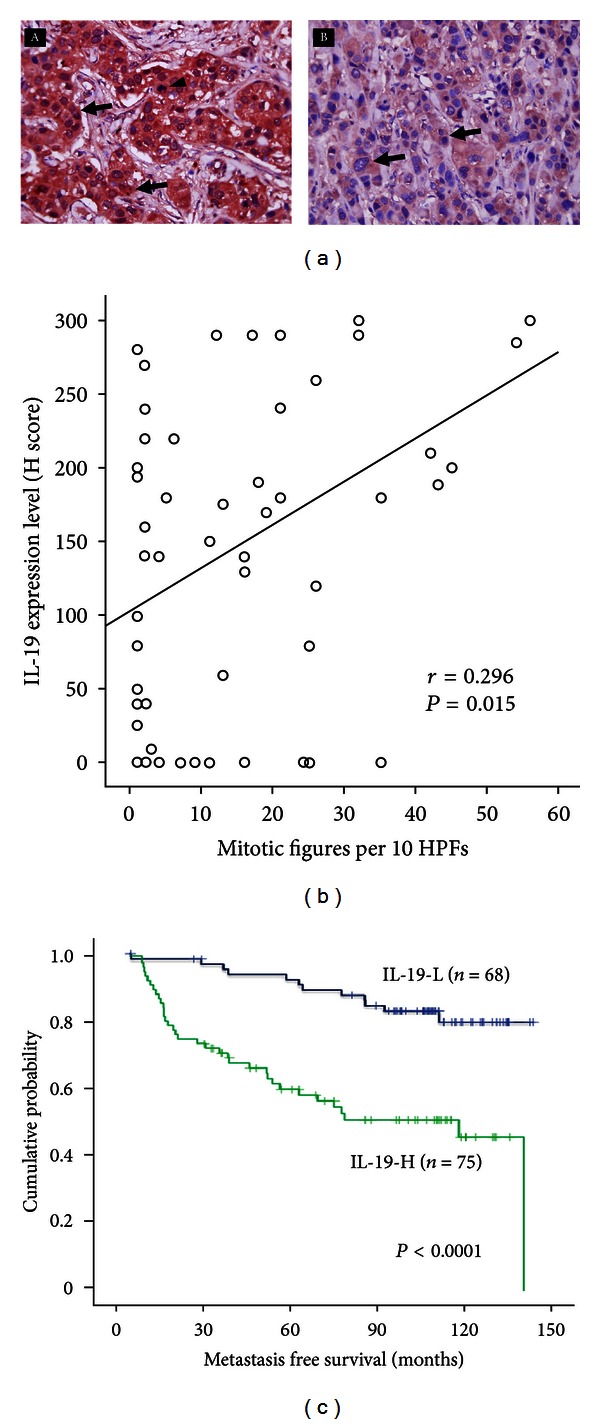
IL-19 expression in breast tumors was correlated with clinical outcome. (a) Immunohistochemical staining (IHC) showed that IL-19 was strongly (A) or weakly (B) stained in breast invasive duct carcinoma (IDC) cells (arrows) (magnification, ×400). Mitotic figures (A, arrowhead) are commonly found in breast cancer cells strongly stained with IL-19. (b) Mitotic figures were correlated with IL-19 expression levels in breast cancer cells. IL-19 expression levels in 60 IDC tissue samples were analyzed using H scoring. HPFs: high power fields. (c) Of the 143 patients from pathology, Kaplan-Meier plots were used to predict the metastasis-free survival based on IL-19 expression levels. The figure refers to Hsing et al. [13].

The most common form of breast cancer, IDC, begins in the cells that grow and form the ducts, and then it invades the fatty tissue outside of the duct and appears as a smooth-edged and hard tumor in the breast [7]. IL-19 expression was substantially greater in breast IDC tissue (Figure 1(a)A) than in healthy tissue (Figure 1(a)B) [13, 22]. IL-19 levels have been verified to be associated with primary tumor status (Table 2), advanced tumor stage, a high degree of occurrence of lymph node and distant metastasis, HER2 status, and the presence of mitotic figures (Figure 1(b)). Higher IL-19 expression levels predicted worse disease-specific survival (Figure 1(c)) and metastasis-free survival with more than 3-fold increased risk (HR = 3.322) (Table 3). However, the serum levels of IL-19 are unchanged with the tumor stage, which suggests that IL-19 levels in the local microenvironment are dominant rather than the systemic effect in breast cancer [13]. Thus, IL-19 expression in tumor cells is hypothesized to be involved in tumor progression and to correlate with the clinical outcome of breast cancer. Thus, IL-19 has the potential to be a prognostic marker for patients with an IDC breast tumor.

## 4. Mechanisms of IL-19 in Breast Cancer

### 4.1. IL-19 Induced Proliferation, Migration, and Fibronectin Expression and Assembly in Breast Cancer Cells, and It Promoted Metastasis

**Figure 2:**
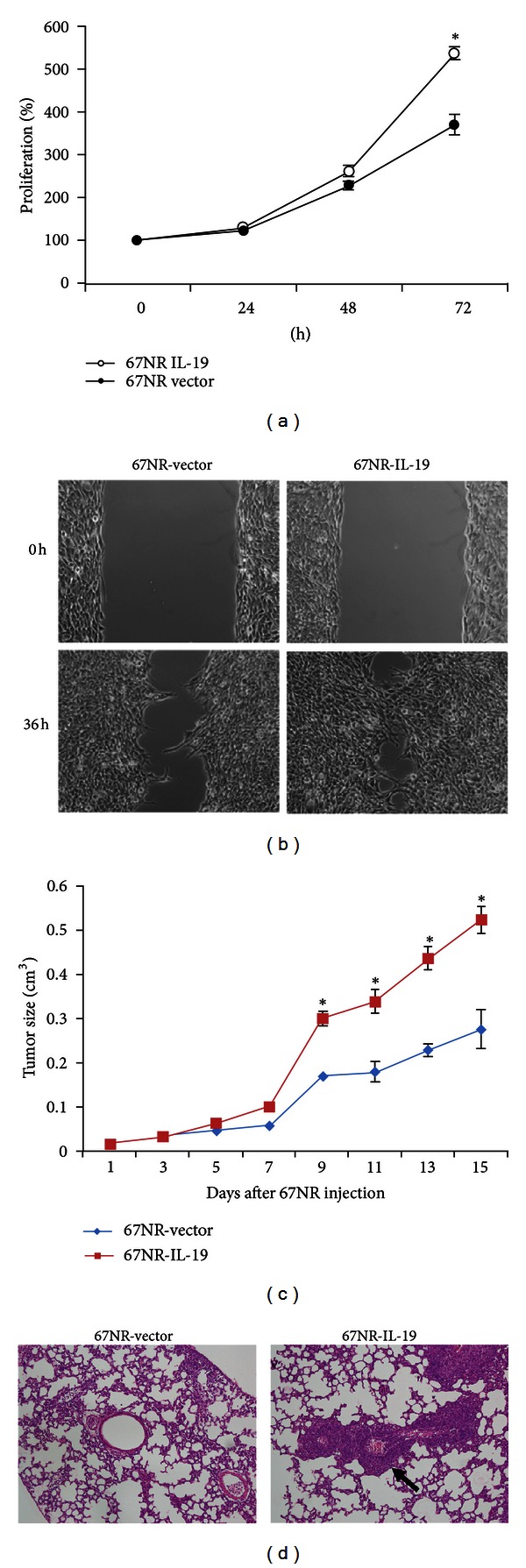
IL-19 overexpression promoted breast cancer cell proliferation and migration *in vitro* and induced tumor growth and metastasis *in vivo*. We constructed the IL-19-overexpressing stable clones of 67NR (67NR/IL-19) cells. (a) Proliferation of 67NR/IL-19 cells was significantly higher than that of control cells. Data are mean ± SEM of triplicate experiments. *P < 0.05 versus control-vector cells. (b) Cell migration, determined using a wound healing assay, was greater in 67NR/IL-19 cells than in control-vector cells. Quantitative results are shown as a wound healing index. Data are mean ± SEM. *P < 0.05 versus control-vector cells. (c) 67NR cells (2 × 10^6^) were injected into the left mammary fat pads of BALB/c mice and tumor growth was determined. (d) Fifteen days later, the tumors were removed. Thirty days after the tumors had been surgically removed, the mice were killed, and histological analysis showed that lung metastasis (arrows) was significantly higher in 67NR/IL-19 cells than in control cells (magnification, ×100). Data are mean ± SEM (*n* = 6 mice in each group). *P < 0.05 versus control mice. The figure refers to Hsing et al. [13].

IL-19 acts in breast tumors in an autocrine manner and the expression and colocalization of IL-19 and its cognate receptors can be seen both in human (MCF-7 and Hs578T) and in mouse (67NR and 4T1) breast cancer cell lines [13]. *In vitro* assays showed that the mechanism of IL-19 in 4T1 cells is activating the intracellular signals STAT3, JNK, ERK, AKT, and NF-*κ*b, which may be involved in cell proliferation and survival, but phosphorylation occurred only on JNK, ERK, and AKT in Hs578T human breast cancer cells [13]. Fibronectin (FN) expression and assembly in tumor cells are well-known promoters of tumor progression and pulmonary metastasis in breast cancer [51]. IL-19 treatment promotes specific proliferation and migration activities as well as fibronectin expression and assembly both in human and in mouse breast cancer cells [13]. In MCF-7 cells, IL-19 directly promotes proliferation, which leads to the increase in the duration of the G2/M stage of the cell cycle [13]. Endogenous fibronectin expression and tumor cell migration are substantiated in IL-19-knockdown 4T1 cells, and treatment of IL-19 further increases migration in IL-19-knockdown cells [13]. Moreover, IL-19 overexpression promotes the proliferation, migration, tumor growth, and metastasis of breast cancer cells (Figure 2).

### 4.2. The Role of IL-19 and CXCR4 during Hypoxia

In most cancers, a principal cause of morbidity and mortality is metastasis, which involves many molecules and numerous interactions between the cancer cells and the host. In breast cancer, hypoxia increases angiogenesis and the release of cytokines, chemokines, and chemokine receptors which are necessary for cancer cell growth and metastasis [52, 53]. CXCR4, one of the well-characterized chemokine receptors expressed in breast cancer cells and attracted by its ligand, SDF-1*α*, is critical in the targeted metastasis of breast cancer [54]. IL-19 induces the expression of CXCR4 in breast cancer cells [13]. A functional hypoxic response element has been found on IL-19 promoter, and hypoxia induces IL-19 expression consistent with the increased expression of CXCR4 in breast cancer cell lines [13]. In response to chemical (CoCl_2_) and physical (hypoxia chamber) hypoxia treatment, anti-IL-19 mAb attenuates hypoxia-induced CXCR4 expression *in vitro* [13]. Therefore, IL-19 may be an upstream molecule that mediates hypoxia-induced CXCR4 expression and metastasis. HER2 increases breast tumor metastasis by promoting the expression and preventing the ligand-induced degradation of CXCR4 [54]. We previously reported a significant clinical correlation between IL-19 and HER2 expression [13]. It would be interesting to explore the relationship between HER2 expression and the hypoxia/IL-19/CXCR4 pathway in the progression of breast cancer.

### 4.3. IL-19 and Extracellular Matrix and Cytokines Network in Breast Cancer

A variety of direct cell-cell, cell-matrix, and paracrine interactions and the release of cytokines are involved in metastasis [55]. With the assistance of polymeric FN assembly [51], breast cancer cells frequently invade other areas of the body, this metastasis is expected to be attenuated when FN-mediated endothelial adhesion is inhibited [56]. The effect of IL-19 on FN expression and assembly in breast cancer cells has been investigated [13]. FN expression was higher in MCF-7 cells, 4T1 cells, and Hs578T cells after IL-19 treatment. Knockdown of endogenous IL-19 in tumor cells inhibited FN expression and assembly, but overexpression of IL-19 induced FN production.

Cytokines may be involved in a network with a large variety of different members that facilitate tumor growth. Matrix metalloproteinase-2 (MMP-2) and IL-6 participate in endothelial cell injury and the extravasation of tumor cells [55], and IL-1*β*, IL-6, and TGF-*β* are involved in tumor progression [7]. In breast cancer, receptor gp130 mediates IL-6 signals; inhibiting IL-6 signaling and production in turn inhibits *in vitro* activation of STAT3 as well as *in vivo* tumor cell proliferation and number of metastases [7]. IL-19 induces IL-1*β*, IL-6, TGF-*β*, and MMP-2 in breast cancer cells. Because stimulation with IL-6 induces IL-19 expression, there may be positive feedback between IL-6 and IL-19 that elicits the STAT3 signal and the progression of breast cancer. It further supports that IL-19 not only directly facilitates cell proliferation, cell migration, and metastasis but it also prompts the expression of IL-1*β*, IL-6, TGF-*β*, MMP-2, MMP9, CXCR4, and fibronectin, which supply of a microenvironment for tumor growth and metastasis [13].

### 4.4. IL-19, Th2 Cytokines, and Tumor-Associated Macrophages

Th2 cytokines—IL-4, IL-13, and IL-10—induce alternatively activated macrophages by increasing macrophage mannose receptor activity [57]. Macrophage infiltration into premalignant mammary tissue was identified during mammary cancer progression in an animal model [58]. Cytokines expressed by Th2 cells regulate the chemoattraction and polarization of tumor-associated macrophages (TAMs) [1]. TAMs have poor antigen-presenting capacity and suppress T-cell activation and proliferation by releasing IL-10, TGF-*β*, and prostaglandins [8, 59]. Th2 cytokines promote M2-polarized phenotype TAMs and are pivotal in subverting adaptive immunity as well as in inflammatory circuits that promote tumor growth and progression [9, 60, 61]. In breast carcinomas, a high level of infiltrating TAMs is associated with a poor prognosis [62].

IL-19 shifts the balance of Th1 and Th2 cells toward Th2 dominance [63–65]. It stimulates secretions of Th2 cytokines (IL-4, -5, -10, and -13) and dose dependently attenuates the secretion of Th1 cytokine (IFN-*γ*) [38, 63]. IL-17 treatment induces IL-19 expression, and the IL-13 and -19 levels are closely correlated with the Th2 response in human asthma [24]. Treating LPS-stimulated monocytes with IL-4 or IL-13 potentiates IL-19 expression [35]. There may be positive feedback between IL-19 and Th2 cytokines. That Th2 cytokines induce the bioactivities of TAMs has been confirmed; however, the association between IL-19 and Th2-polarized TAMs remains to be clarified.

## 5. Conclusions

**Figure 3:**
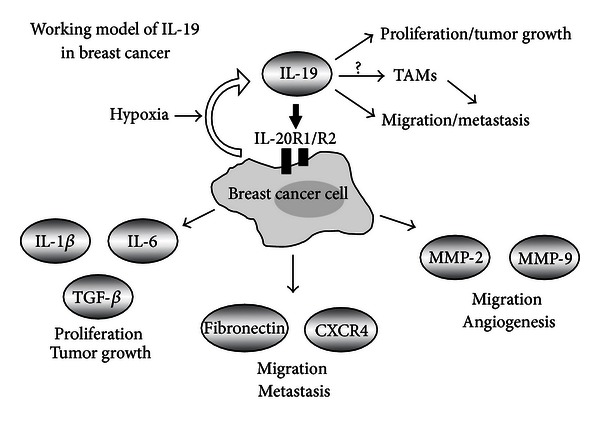
A schematic diagram which shows that IL-19 acts in an autocrine manner. Hypoxia also induces IL-19 production. IL-19 directly promotes the proliferation and migration of breast cancer cells. In addition, IL-19 indirectly induces tumor progression including angiogenesis, tumor growth, and metastasis through MMP2, MMP9, IL-1*β*, IL-6, TGF-*β*, CXCR4, and fibronectin. The hypothesized working model of IL-19 in breast cancer.

Breast cancer development and progression are known to be affected by the dynamic interplay between microenvironmental effectors and the immune responses and intrinsic properties of the tumor cells. Accumulated clinical and experimental data indicate that IL-19 is an important mediator in breast cancer. The consequences of IL-19 expression in breast cancer development and progression are as follows: (a) it affects the clinical outcome of breast cancer and tumor metastasis; (b) it increases cell proliferation, cell migration, and fibronectin assembly; (c) hypoxia induces IL-19-mediated gene-expression in cancer cells, which promotes proliferation (IL-1*β*, IL-6, and TGF-*β*), migration or metastasis (MMP2, MMP9, CXCR4, and fibronectin), or angiogenesis (MMP2 and MMP9); (d) it facilitates tumor growth and lung metastasis.  Figure 3 shows a hypothetical working model related to the pathogenesis of tumor growth in breast cancer to explain the role of IL-19 in the pathogenesis of tumor progression. We conclude that IL-19 has an autocrine effect on breast cancer cells and provides a microenvironment for tumor progression. Antagonizing IL-19 might have therapeutic potential in breast cancer.

## Figures and Tables

**Table 1:** Tumor cells stained for IL-19 in neoplastic tissue.

Tumor Type	IL-19 Immunostaining
(1) Skin, SCC	+
(2) Buccal mucosa, SCC	++
(3) Tongue, SCC	++
(4) Esophagus, SCC	++
(5) Lung, SCC	+
(6) Breast, IDC	++
(7) Liver, HCC	+
(8) Kidney, RCC	+
(9) Ovary, clear cell carcinoma	+
(10) Bladder, TCC	+/−
(11) Thyroid, papillary carcinoma	+/−
(12) Thymus, thymic carcinoma	+/−
(13) Lymph node, B cell lymphoma	+/−
(14) Stomach, adenocarcinoma	−
(15) Colon, adenocarcinoma	−

++: strongly stained; +: moderately stained; +/−: weakly stained; −: not stained.

SCC: squamous cell carcinoma; IDC: infiltrating duct carcinoma; HCC: hepatocellular carcinoma; RCC: renal cell carcinoma; TCC: transitional cell carcinoma.

The table refers to Hsing et al. [22].

**Table 2:** Associations between IL-19 expression in 143 breast invasive duct carcinoma tumors with other important clinicopathologic variables.

Parameters	Category	*n*	IL-19-L^1^	IL-19-H^2^	*P*
			(*n* = 68)	(*n* = 75)	
Age (years)	<60	103	54	49	0.061
	≥60	40	14	26	

Primary tumor (T)	T1	50	33	17	0.003*
	T2	78	32	46	
	T3	10	3	7	
	T4	5	0	5	

Nodal status (N)	N0	71	42	29	0.001*
	N1	56	25	31	
	N2	16	1	15	

Stage	I	34	24	10	<0.001*
	II	87	41	46	
	III	22	3	19	

HER2 expression	Low (0+ to 2+)	103	56	47	0.009*
	High (3+)	40	12	28	

^1^IL-19-L: low grade immunostaining, *H* score <200.

^2^IL-19-H: high grade immunostaining, *H* score ≥200.

^3^HER scoring was done using standard HercepTest guidelines.

*Statistically significant.

The table refers to Hsing et al. [13].

**Table 3:** Multivariate survival analyses for MFS.

Parameter	Category	MFS		
		HR	95% CI	*P *
IL-19 expression	Low (*H* < 200)	1	—	0.0004*
	High (*H* ≥ 200)	3.322	1.711–6.453	
Stage	I	1	—	0.0079*
	II-III	4.961	1.522–16.178	

MFS: metastasis free survival.

HR: hazard ratio.

*Statistically significant.

The table refers to Hsing et al. [13].
